# Safety in the Age of Artificial Intelligence: Evaluating Large Language Model Adherence to Antithrombotic Medication and Regional Anesthesia Guidelines

**DOI:** 10.7759/cureus.114071

**Published:** 2026-08-06

**Authors:** Conner M Willson, Birpartap S Thind, Jay Srinivas, Anita Gupta

**Affiliations:** 1 Department of Anesthesiology, Loma Linda University Medical Center, Loma Linda, USA; 2 Department of Anesthesiology, Riverside Community Hospital, Riverside, USA; 3 Global Policy and AI, Independent Researcher, San Diego, USA; 4 Department of Anesthesiology and Critical Care Medicine, Johns Hopkins University School of Medicine, Baltimore, USA; 5 Department of Anesthesiology, University of California Riverside School of Medicine, Riverside, USA

**Keywords:** artificial intelligence in medicine, clinical decision support, large language models (llm), neuraxial anesthesia, pain management guidelines, patient safety, regional anesthesia and pain management

## Abstract

The release of the 2025 American Society of Regional Anesthesia (ASRA) 5th edition guidelines for regional and neuraxial anesthesia procedures for patients receiving antithrombotic medications introduced complex, patient-specific hold times and resumption protocols. As clinicians increasingly utilize large language models (LLMs) as clinical decision support tools, the reliability of these models remains largely unvalidated. This study evaluates the accuracy of two of the foremost LLMs, ChatGPT (OpenAI, San Francisco, CA) and Google Gemini (Google DeepMind, London, UK), in adhering to these new gold-standard safety guidelines.

Twenty-five standardized clinical vignettes were developed. Each vignette featured a patient on a specific anticoagulant (e.g., rivaroxaban, apixaban, dabigatran) requiring a neuraxial or regional anesthetic procedure (stratified by high-risk vs. low-risk). Variables included renal function, dose frequency, and procedural urgency. Prompts were submitted to the latest publicly available ChatGPT and Google Gemini models with separate instructions to provide hold and resumption times. LLMs were queried to ensure familiarity with 2025 ASRA guidelines prior to submission of prompts. Responses were graded against the 2025 ASRA guidelines by independent reviewers. Response adherence was categorized as: 1. concordant (100% match); 2. conservative error (LLM recommended time was longer than required); 3. dangerous error (recommended time was shorter than required, a critical safety violation); or 4. omission (no specific timeframe provided).

ChatGPT achieved a concordance rate of 64%, compared to Gemini at 62%. However, the models displayed distinct error profiles. ChatGPT produced "dangerous errors" in 20% of evaluations and failed to specify a time in 16% of cases. In contrast, Gemini’s dangerous error rate was lower at 12%, but it demonstrated a significant "conservative error" rate of 22%, compared to 0% for ChatGPT. A chi-square test indicated that the difference in dangerous error rates between the two models was not statistically significant. Regardless, notable differences in error character and response completeness were observed. Gemini was more consistent in providing specific timeframes in 96% of prompts compared to 84% for ChatGPT. Variability of responses between users was determined via a chi-square test of homogeneity and was found to be statistically significant only for Gemini.

While both models demonstrated moderate guideline awareness, their failure modes differed meaningfully. ChatGPT's errors were predominantly dangerous underestimations and omissions, while Gemini exhibited a conservative bias, overestimating hold times when a match was not achieved. Significant limitations exist regarding generalizability of these results. Only two major LLM models were tested. Other, more clinically oriented models exist, and newer versions of both ChatGPT and Gemini are consistently being released. These may improve LLM adherence to clinical guidelines. Despite these limitations, this study highlights the dangers of utilizing LLMs for periprocedural anticoagulation decision-making in regional and neuraxial anesthesia. Both models produced potentially dangerous recommendations in 12-20% of scenarios. Ongoing evaluation of LLM adherence to evolving guidelines remains essential, and clinicians must exercise extreme caution when utilizing these tools in patient care.

## Introduction

Neuraxial interventions, including epidural and spinal procedures, carry an inherent risk of dangerous hemorrhage in patients receiving anticoagulant therapy. Because the spinal canal is a confined, non-compressible space, hemorrhagic complications often result in irreversible neurological injury. This creates a critical clinical dilemma of managing the risk of procedural bleeding versus the consequences of temporary therapy cessation [1-2]. Even though the overall risk of major bleeding with neuraxial procedures is small, the consequences are catastrophic when they occur, while the role of antithrombotic therapy in preventing acute cerebral and cardiovascular events is equally crucial [2]. Patients maintained on anticoagulant or antiplatelet therapy increasingly present for procedures requiring regional or neuraxial techniques, whether in the perioperative setting or interventional pain procedures. 

Antithrombotic medications serve critical functions, including the prevention of venous thromboembolism in high-risk immobilized or hospitalized patients, as well as the reduction of arterial thrombotic risk in those with coronary artery disease or prior cerebrovascular events. Interruption is therefore never without risk. Layered on top of this procedural complexity is the constantly evolving pharmacologic landscape for these medications. While older medications such as warfarin and unfractionated heparin are well characterized and have strongly defined monitoring guidelines, newer medications such as direct oral anticoagulants (DOACs) present a different set of challenges. Dabigatran, rivaroxaban, apixaban, and related agents have become first-line therapy for atrial fibrillation and venous thromboembolism, and a growing number of indications since their FDA approvals between 2010 and 2012 [2-3]. They offer meaningful advantages over warfarin in terms of dosing simplicity and monitoring burden for long-term use, but these same elements conversely create a more difficult periprocedural decision-making process [2]. Renal function, dosing frequency, and drug-specific half-lives interact in ways that make the appropriate hold time genuinely patient-specific, preventing reduction to a single universal protocol [3].

The American Society of Regional Anesthesia and Pain Medicine (ASRA) has worked to address this complexity through successive guideline revisions. The 2025 fifth edition of the ASRA guidelines is the most significant update the guidelines have seen since the specialty-specific framework was first introduced [4]. Among the most clinically important changes, the new guidelines distinguish between low-dose and high-dose DOAC regimens and introduce specific drug-level thresholds for procedural safety, specifying that residual apixaban, rivaroxaban, or edoxaban levels below 30 ng/mL, or anti-Xa activity at or below 0.1 IU/mL, are acceptable prior to neuraxial block, deep plexus block, or peripheral nerve block [4,5]. This represents a meaningful shift away from time-based protocols and toward pharmacokinetically informed, individualized decision-making. For clinicians opting for the threshold-based pathway, this requires integrating drug class, dose, dosing frequency, renal function, and a laboratory result into a single periprocedural recommendation.

This complexity makes the growing informal use of large language models (LLMs) in clinical practice particularly worth examining. Tools such as ChatGPT (OpenAI, San Francisco, CA) and Google Gemini (Google DeepMind, London, UK) have moved quickly from novelty to routine in many clinical environments, with applications across decision support, documentation, education, and patient communication. The volume of research evaluating LLMs in medicine has been remarkable, growing from a single relevant publication in 2019 to over 550 in 2024 [6,7]. A 2026 survey by the American Medical Association found that 81% of physicians now use AI tools professionally, more than double the rate reported in 2023, with the most common application being the review and summarization of medical research and clinical standards of care [8]. Many clinicians are already using these tools to look up medication dosing, review guideline recommendations, and obtain rapid answers to clinical questions, including questions about periprocedural anticoagulation management.

The concern is that these models are not yet validated for accuracy nor transparent about their limitations. Research has shown that despite strong performance on standardized medical examinations, LLMs perform significantly worse on assessments that require dynamic clinical reasoning, specifically the ability to update a management recommendation when new patient information changes the picture [9]. Their outputs can be confidently stated even when incorrect, and they rarely provide the reasoning or source behind a recommendation [6]. In the context of the 2025 ASRA guidelines, with their nuanced, patient-specific DOAC protocols integrating dose, renal function, procedure risk tier, and drug-level thresholds, that combination of apparent confidence and potential inaccuracy carries real patient safety implications.

This study was designed to evaluate how well ChatGPT and Google Gemini perform against the 2025 ASRA guidelines in exactly this clinical context. Our interest was not only in whether the models arrived at the correct answer, but in the character of their errors. Specifically, whether incorrect responses were conservative or potentially dangerous, and whether the models were willing to commit to a specific time-based recommendation at all.

## Materials and methods

This was a prospective comparative observational study assessing the accuracy of LLMs in providing anticoagulation management recommendations consistent with the 2025 ASRA guidelines for regional and neuraxial anesthesia in patients receiving antithrombotic therapy. Twenty-five standardized fictitious clinical vignettes were developed by experts in the field of anesthesiology and pain medicine, representing the most commonly encountered clinical scenarios within clinical practice. Vignettes were created by experts in the field of anesthesiology and pain medicine. The scenarios involved fictional patients receiving specific antithrombotic agents periprocedurally and tested the LLM's response regarding use of these agents for a given regional or neuraxial anesthetic procedure. Each vignette was tested for appropriate cessation time prior to regional/neuraxial anesthesia and appropriate resumption time following the procedure. The vignettes aimed to fully cover the scope of antithrombotic medications and clinical scenarios addressed by the 2025 ASRA guidelines.

Vignettes were designed to vary across four key clinical dimensions that are known to influence periprocedural anticoagulation management under the 2025 ASRA guidelines. First, antithrombotic agent and dose frequency were varied, with some vignettes specifying once-daily dosing and others twice-daily dosing. Second, renal function was incorporated as a variable, with creatinine clearance values ranging from normal to severely impaired, given the renal-dependent clearance of certain antithrombotic medication and their effects on recommended hold durations. Third, procedure risk stratification was applied across the vignette set, with procedures classified as either low-risk or high-risk in accordance with the ASRA framework. Fourth, procedural urgency was included as a variable in select vignettes to assess model behavior in time-sensitive clinical scenarios.

Two LLM platforms were selected for evaluation: ChatGPT, developed by OpenAI, and Google Gemini, developed by Google. Both platforms were selected on the basis of their widespread availability to clinicians without institutional credentialing or subscription, reflecting their real-world pattern of use as informal clinical reference tools. Both models were accessed utilizing their web interface. The latest available models of each LLM at the time of the data collection period (March 2026), ChatGPT 5.3 and Google Gemini 3.1, were utilized. The length of the data collection phase was limited to a single week to reduce any changes. Each model was queried with 25 vignette prompts, once to determine cessation time and again to determine resumption time, for a total of 50 responses per LLM, per reviewer. Each of the three reviewers collected their own set of data separately, yielding a total sample size of 300 responses (150 for ChatGPT, 150 for Gemini). 

To minimize the potential influence of prior user-specific query history, personalization data, and browser-based tracking on model responses, all queries were conducted using Brave browser in “private” mode. All queries were performed by each of the three reviewers, all at different locations, with different internet protocol (IP) addresses, and on different devices. Each reviewer was initially blinded to the data from other reviewers until all data collection was complete. After completion of data collection, all reviewers independently reviewed the entirety of the LLM queries and responses and checked for appropriate grading. 

Prior to submission of the clinical vignettes, each model was queried to assess its self-reported familiarity with the 2025 ASRA guidelines, including whether the model acknowledged awareness of the fifth edition and its key updates. This preliminary query was not scored but was used to document baseline model awareness of the relevant guideline version before proceeding with vignette submission. All vignettes were submitted using a zero-shot prompting approach, meaning no examples of correct responses were provided to the model prior to each query, and no chain-of-thought or step-by-step reasoning instructions were included [10]. 

**Figure 1:**
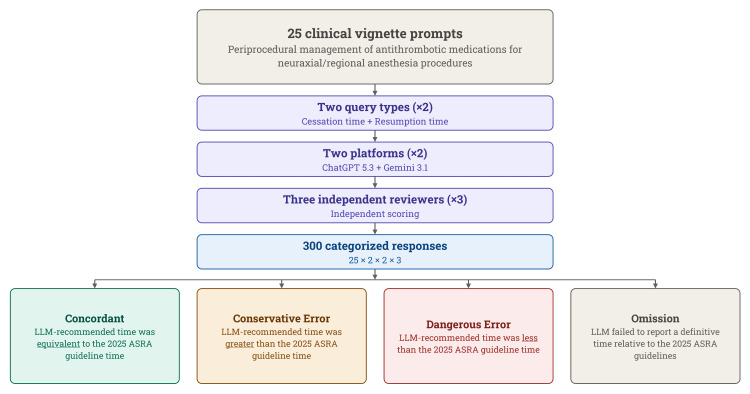
Methodology flowchart and categorization of LLM responses LLM = Large Language Model; ASRA = American Society of Regional Anesthesia

Each response was assigned to one of four categories. A response was classified as “concordant” if both the hold time and the resumption time matched the guideline recommendation exactly. A response was classified as a “conservative error” if the recommended hold time exceeded what the guidelines specify, representing an overly cautious recommendation that could result in unnecessary procedure delay or prolonged interruption of anticoagulation. A response was classified as a “dangerous error” if the recommended hold time was shorter than what the guidelines specify, representing the highest-risk error category with direct potential for patient harm. Any response in which the model declined to provide a specific time-based recommendation or stated that it could not answer the question was recorded separately as an “omission” (Figure 1).

Concordance rates and error category frequencies were calculated as proportions for each model. The primary outcome was to determine the "dangerous error" rates for each LLM in regards to 2025 ASRA antithrombotic medication guidelines for regional and neuraxial anesthesia procedures. The secondary outcomes were to evaluate the differences in LLM error types and variability in LLM responses. Differences in response types between LLMs were assessed using a chi-square test. Reviewer responses were additionally utilized to calculate a chi-square test of homogeneity to assess how LLMs responses may vary user-by-user. A p-value of less than 0.05 was considered statistically significant.

## Results

**Figure 2:**
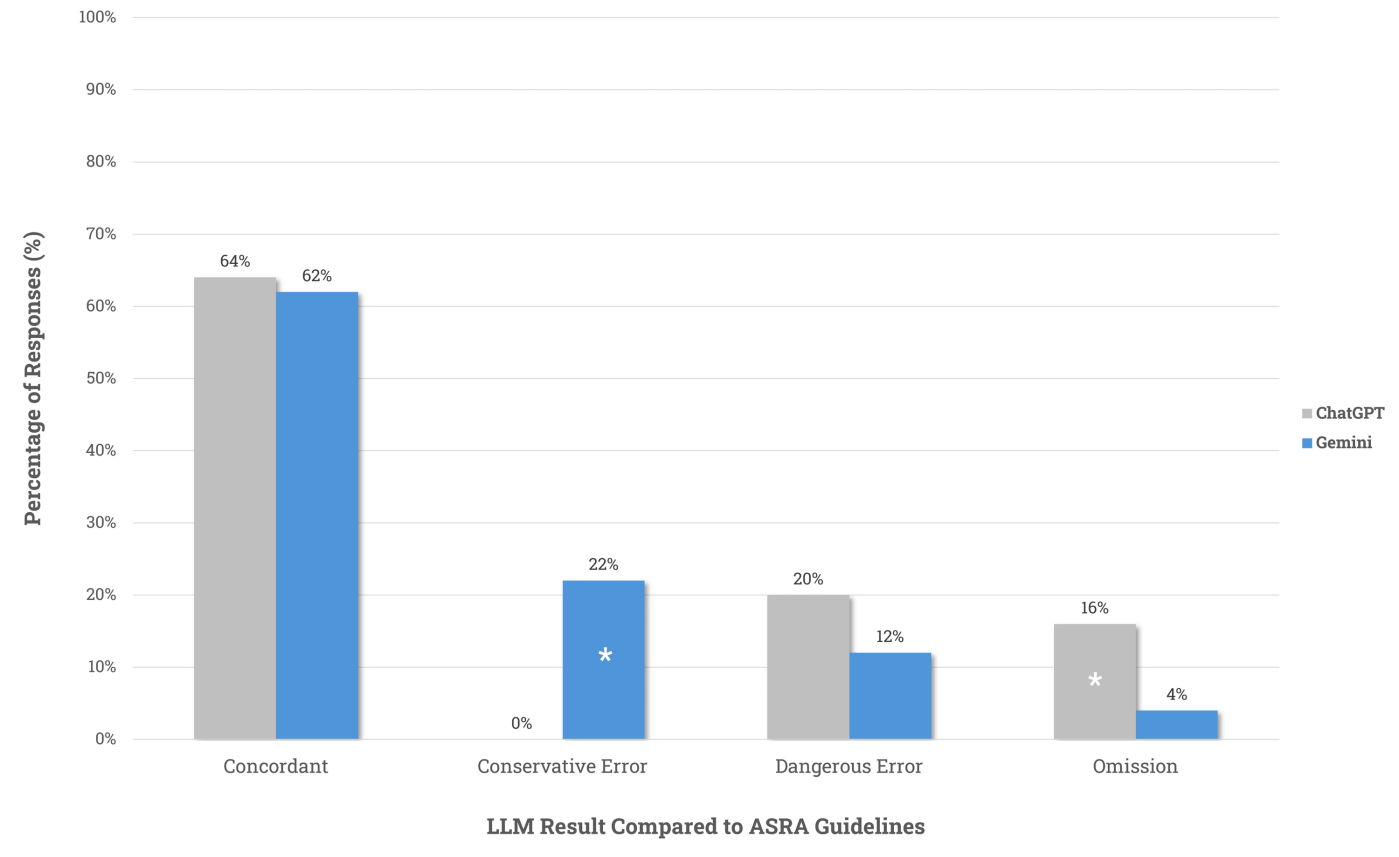
Comparison of response accuracy between ChatGPT and Google Gemini across 50 evaluated prompts per model, displayed in percentages A chi-square test was performed for each category, with a p-value of <0.05 considered significant. There was no statistically significant difference in concordant results between ChatGPT and Gemini LLMs (χ² = 0.054, p = 0.836). There was a statistically significant difference (denoted by asterisk) in conservative error rate, with Gemini having an error rate of 22% compared to ChatGPT at 0% (χ² = 12.36, p = 0.0004). There was no statistically significant difference in dangerous error rates between ChatGPT and Gemini (χ² = 1.19, p = 0.275). Omissions were far more common with ChatGPT, and were considered statistically significant (χ² = 4.00, p = 0.046).

Across 50 evaluated responses per model and per reviewer, ChatGPT achieved a concordance rate of 64% (32/50) and Gemini achieved 62% (31/50), reflecting broadly similar performance in terms of exact guideline adherence. Concordance rates between both LLMs were determined not to be statistically significant via chi-square analysis (χ² = 0.054, p = 0.836). Despite this comparable overall accuracy, the two models demonstrated markedly different error profiles when responses deviated from the guideline standard (Figure 2).

Both LLMs were overall error prone. Gemini produced a considerable number of conservative errors at 22% (11/50), while ChatGPT did not produce any (0%, 0/50). This result was determined to be significant (χ² = 12.36, p = 0.0004). In regards to dangerous errors, which have a greater likelihood of causing devastating complications, ChatGPT produced more compared to Gemini, occurring in 20% of evaluations (10/50) compared to 12% (6/50). This was not determined to be statistically significant (χ² = 1.19, p = 0.275). 

Omission rates between the two LLMs also differed greatly, with a rate of 16% for ChatGPT (8/50) compared to a rate of 4% for Gemini (2/50). This was determined to be statistically significant (χ² = 4.00, p = 0.046). Gemini’s much larger conservative error rate may explain the lower omission rate, therefore the relevance of this finding is likely minimal as a result.

Response variability between users for each LLM was evaluated via a chi-square test of homogeneity. Homogeneity in responses between reviewers was not statistically significant for ChatGPT (χ² = 8.14, p = 0.087), but was statistically significant for Gemini (χ² = 17.62, p = 0.0073). This indicates that LLM responses did not meaningfully differ for each reviewer with ChatGPT, but did with Gemini. 

## Discussion

ChatGPT and Gemini LLMs possess some degree of familiarity with the 2025 ASRA guidelines, achieving concordance rates of 64% and 62% respectively. However, the concordance rate alone does not adequately characterize the clinical utility or safety of these tools. The more revealing finding lies in the divergent character of each model's errors, which differed substantially and in ways that carried meaningful implications for patient safety.

ChatGPT's failure mode was predominantly one of underestimation and omission. In 20% of evaluations, recommendations for hold times shorter than the guideline standard were produced, and in a further 16% it declined to provide a specific timeframe at all. In the context of periprocedural anticoagulation management, a dangerous error is not simply an academic discrepancy. This error type represents a recommendation for which, if followed, could result in a regional or neuraxial procedure being performed before adequate drug clearance, with the potential for severe complications [1]. ChatGPT produced no conservative errors, revealing that when it deviates from the guideline, it does so consistently in the more hazardous direction.

Gemini's error profile was structurally different. Its dangerous error rate of 12% was lower than ChatGPT's, and it produced omissions in only 4% of cases, reflecting a greater willingness to commit to a specific recommendation. However, Gemini demonstrated a conservative error rate of 22%, recommending hold times exceeding guideline specifications in nearly one in four responses. While conservative errors do not carry the same immediate hemorrhagic safety risk as dangerous ones, they are not benign. Unnecessarily prolonged interruption of anticoagulant therapy increases the risk of thromboembolic events, and in patients on antiplatelet therapy for coronary artery disease or prior cerebrovascular events, the risk of arterial thrombosis during an extended hold period is clinically significant [2,11]. Furthermore, conservative errors may also result in avoidable procedure delays with downstream implications for patient care and resource utilization.

While the difference in dangerous error rates between the two LLMs was not determined to be statistically significant, the overall error rate and presence of a non-insignificant number of dangerous errors from both models should be seen as very concerning. Clinicians utilizing these tools need to proceed with extreme caution, and should be utilizing trusted resources to verify LLM responses. Recent literature has demonstrated high error rates across all common LLMs, especially for differential diagnosis generation [12]. LLMs come at high risk for inaccuracies, such as hallucinations, old and inaccurate training data, and training based on data from unreputable sources [12]. LLM algorithms, especially those that fail to use retrieval-augmented generation (RAG), which directs the LLM to verify with trusted resources while generating a response, show strong failure rates with medical guideline management [13-14]. RAG-enhanced LLMs generally perform better for guideline-directed questions [15]. While newer models and guideline-directed questions typically prompt common LLMs like ChatGPT and Gemini to utilize RAG, its utilization status is not immediately or easily known by the user unless a specific model is designed. While RAG use does reduce hallucinations and provide improved accuracy, LLMs tend to reduce RAG use when possible due to increased lag in response time [16]. Both ChatGPT and Gemini models reveal this practice when queried about their RAG use. With this in mind, it is reasonable to assume that poor prompt phrasing and use of free and older models may reduce LLM adherence for guideline questions. These two newer model LLMs tested in this study demonstrated a very high error rate despite prompting to recall the 2025 ASRA guidelines specifically prior to queries. 

The growing informal use of these tools by clinicians makes these findings particularly relevant. A 2026 AMA survey found that 81% of physicians now use AI tools professionally, with the most common application being the review and summarization of medical research and clinical standards of care [8]. It is reasonable to assume that a proportion of these queries involve periprocedural high-stakes medication management, including antithrombotic medication hold times. Without validated performance data, clinicians have no basis for calibrating their trust in these responses at this time. LLMs present guideline-like confidence in their responses, which can lead to false trust, even if recommendations are frequently incorrect [17]. Without distinct knowledge of the inner workings of the specific LLM in use, it is relatively easy for clinicians to be misled by LLM responses.

This study has several significant limitations. Most notable is the limited set of clinical vignettes and LLM models used. Expansion to a larger set may better reveal LLM tendencies when it comes to these specific guidelines. Utilizing a wider array of LLMs would be helpful in understanding which LLMs are superior at retrieving clinical guideline data. A higher reviewer count would additionally improve the accuracy of data variability assessments. Another major consideration is that it is difficult to recreate the typical experience that a clinician may have utilizing these tools. This is especially notable given Gemini's wide variation in responses observed amongst the three reviewers. While this study intentionally blinded each LLM to prior user history, clinicians will likely be using an account tied to a history of asking clinical questions, which may influence responses. In addition, LLMs are rapidly evolving, with new models being released frequently. Newer models are expected to have improved abilities to collect and review data. This study was designed to evaluate what was believed to be the most common experience users would have when asking questions regarding antithrombotic medication guidelines for regional and neuraxial anesthetic procedures. 

As LLMs continue to enhance and become more integrated with clinical care, future research will be necessary to continually assess their efficacy and shortcomings regarding important clinical decisions. Future research into this topic should include expansion into all types of LLMs, use of custom RAG-based LLMs, and exploration of more topics related to anesthesiology and pain medicine. These studies will be crucial in helping clinicians better understand the considerations of using these tools as adjuncts for direct patient care. 

## Conclusions

Currently, commonly used LLMs cannot be relied upon as standalone references for periprocedural anticoagulation decision-making in regional and neuraxial anesthesia, specifically regarding the 2025 ASRA guidelines. Two distinct error profiles were discovered. ChatGPT more frequently suggested potentially dangerous underestimations, while Gemini typically favored excessive caution. Regardless, given the total level of errors made, neither LLM tested should be seen as acceptable for trusting in regard to patient care. While other LLMs were not tested, clinicians should be extremely cautious about trusting any model until it is well validated on the topic they are requesting assistance with. As LLMs continue to progress and evolve, future research will be required for ongoing evaluation of their trustworthiness.
