# Correction: Safety in the Age of Artificial Intelligence: Evaluating Large Language Model Adherence to Antithrombotic Medication and Regional Anesthesia Guidelines

**DOI:** 10.7759/cureus.c472

**Published:** 2026-08-17

**Authors:** Conner M Willson, Birpartap S Thind, Jay Srinivas, Anita Gupta

**Affiliations:** 1 Department of Anesthesiology, Loma Linda University Medical Center, Loma Linda, USA; 2 Department of Anesthesiology, Riverside Community Hospital, Riverside, USA; 3 Global Policy and AI, Independent Researcher, San Diego, USA; 4 Department of Anesthesiology and Critical Care Medicine, Johns Hopkins University School of Medicine, Baltimore, USA; 5 Department of Anesthesiology, University of California Riverside School of Medicine, Riverside, USA

This article has been corrected to update the affiliation for Jay Srinivas to: Global Policy and AI, Independent Researcher, San Diego, USA.

